# A CT-based machine learning model for using clinical-radiomics to predict malignant cerebral edema after stroke: a two-center study

**DOI:** 10.3389/fnins.2024.1443486

**Published:** 2024-10-03

**Authors:** Lingfeng Zhang, Gang Xie, Yue Zhang, Junlin Li, Wuli Tang, Ling Yang, Kang Li

**Affiliations:** ^1^North Sichuan Medical College, Nanchong, China; ^2^Department of Radiology, The Third People's Hospital of Chengdu, Chengdu, China; ^3^Department of Radiology, Chongqing General Hospital, Chongqing, China; ^4^Chongqing Medical University, Chongqing, China

**Keywords:** ischemic, malignant cerebral edema, radiomics feature, machine learning, prediction

## Abstract

**Purpose:**

This research aimed to create a machine learning model for clinical-radiomics that utilizes unenhanced computed tomography images to assess the likelihood of malignant cerebral edema (MCE) in individuals suffering from acute ischemic stroke (AIS).

**Methods:**

The research included 179 consecutive patients with AIS from two different hospitals. These patients were randomly assigned to training (*n* = 143) and validation (*n* = 36) sets with an 8:2 ratio. Using 3DSlicer software, the radiomics features of regions impacted by infarction were derived from unenhanced CT scans. The radiomics features linked to MCE were pinpointed through a consistency test, Student’s t test and the least absolute shrinkage and selection operator (LASSO) method for selecting features. Clinical parameters associated with MCE were also identified. Subsequently, machine learning models were constructed based on clinical, radiomics, and clinical-radiomics. Ultimately, the efficacy of these models was evaluated by measuring the operating characteristics of the subjects through their area under the curve (AUCs).

**Results:**

Logistic regression (LR) was found to be the most effective machine learning algorithm, for forecasting the MCE. In the training and validation cohorts, the AUCs of clinical model were 0.836 and 0.773, respectively, for differentiating MCE patients; the AUCs of radiomics model were 0.849 and 0.818, respectively; the AUCs of clinical and radiomics model were 0.912 and 0.916, respectively.

**Conclusion:**

This model can assist in predicting MCE after acute ischemic stroke and can provide guidance for clinical treatment and prognostic assessment.

## 1 Introduction

AIS is widely acknowledged as the primary cause of mortality and impairment among adults (Meschia and Brott, 2018). Cerebral edema is a frequent complication after AIS and may be linked to poor outcomes. According to the guidelines of the “Safe Implementation of Thrombolysis in Stroke-Monitoring Study (SITS-MOST) program” (Thorén et al., 2017), cerebral edema can be classified into three levels: CED-1 denotes the moment when swelling in brain tissue extends to one-third of the brain’s hemisphere; CED-2 occurs when there is swelling covering over a third of the hemisphere but no midline displacement has occurred; and CED-3 occurs when the enlargement of brain tissue results in the displacement of the midline. Usually, a midline shift (MLS) greater than 5 mm is considered an indicator of malignant cerebral edema (MCE) (Barber et al., 2003). This is considered an indicator of malignant cerebral edema (MCE), which has a mortality rate of up to 80%. Neurological function typically deteriorates rapidly within 2 to 3 days after symptom onset (Liebeskind et al., 2019). Early decompression by debulking flaps can effectively reduce mortality (Minnerup et al., 2011). Prompt identification of the MCE is vital for stroke neurologists to proactively intervene to avert deterioration of the condition and decide on treatments.

To date, MCE can be predicted by various methods, such as the National Institutes of Health Stroke Scale (NIHSS) score, stroke size, Alberta stroke program early CT score (ASPECTS), collateral circulation score, net water uptake (NWU), and cerebrospinal fluid (CSF) displacement (Thomalla et al., 2003; MacCallum et al., 2014; Kim et al., 2015; Dhar et al., 2016; Jo et al., 2017; Nawabi et al., 2021). Currently, non-contrast computed tomography (NCCT) images are recommended by guidelines as the preferred examination for AIS (Powers et al., 2019). However, diffusion-weighted imaging (DWI), fluid-attenuated inversion recovery (FLAIR) imaging, and computed tomography perfusion (CTP) have also been developed as screening modalities for patients with suspected AIS. Although CTP has gradually gained popularity, it is time-consuming and has contraindications for screening.

In recent years, advances in computer technology have enabled the conversion of imaging data into high-throughput information, which can indirectly respond to heterogeneity at the microscopic level of tissue. Texture feature-based radiomics methods are now commonly used (Lin et al., 2020; Xu et al., 2020; Ren et al., 2023). However, it is not entirely clear how NCCT infarct region-based radiomics features correlate with the progression of MCE after AIS. Recently, advancements in artificial intelligence technology have resulted in the steady integration of machine learning (ML) into AIS-related research. This is due to its superior modeling performance over traditional statistical techniques (Hoffman et al., 2023). To date, ML has been used to achieve automatic segmentation of CSF after AIS as a way to predict the risk of MCE and has achieved good results (Dhar et al., 2018). While research has focused on forecasting MCE risk post-AIS using MRI radiomics, NCCT radiomics based on the middle cerebral artery territory, and ML models derived from clinical data (Wang et al., 2021; Hoffman et al., 2023; Wen et al., 2023), the integration of clinical and radiomics data in ML models for predicting MCE post-AIS remains undocumented. Our research led to the creation and validation of ML algorithms that merge clinical and NCCT infarct region radiomics features to predict MCE risk post-AIS. It was hypothesized that machine learning could accurately predict MCE risk post-AIS using both clinical and radiomic data.

## 2 Materials and methods

### 2.1 Patients

This retrospective analysis received approval from our institutional review board (approval number: KY S2023-077-01), and informed consent was waived because of its retrospective nature.

**Figure 1:**
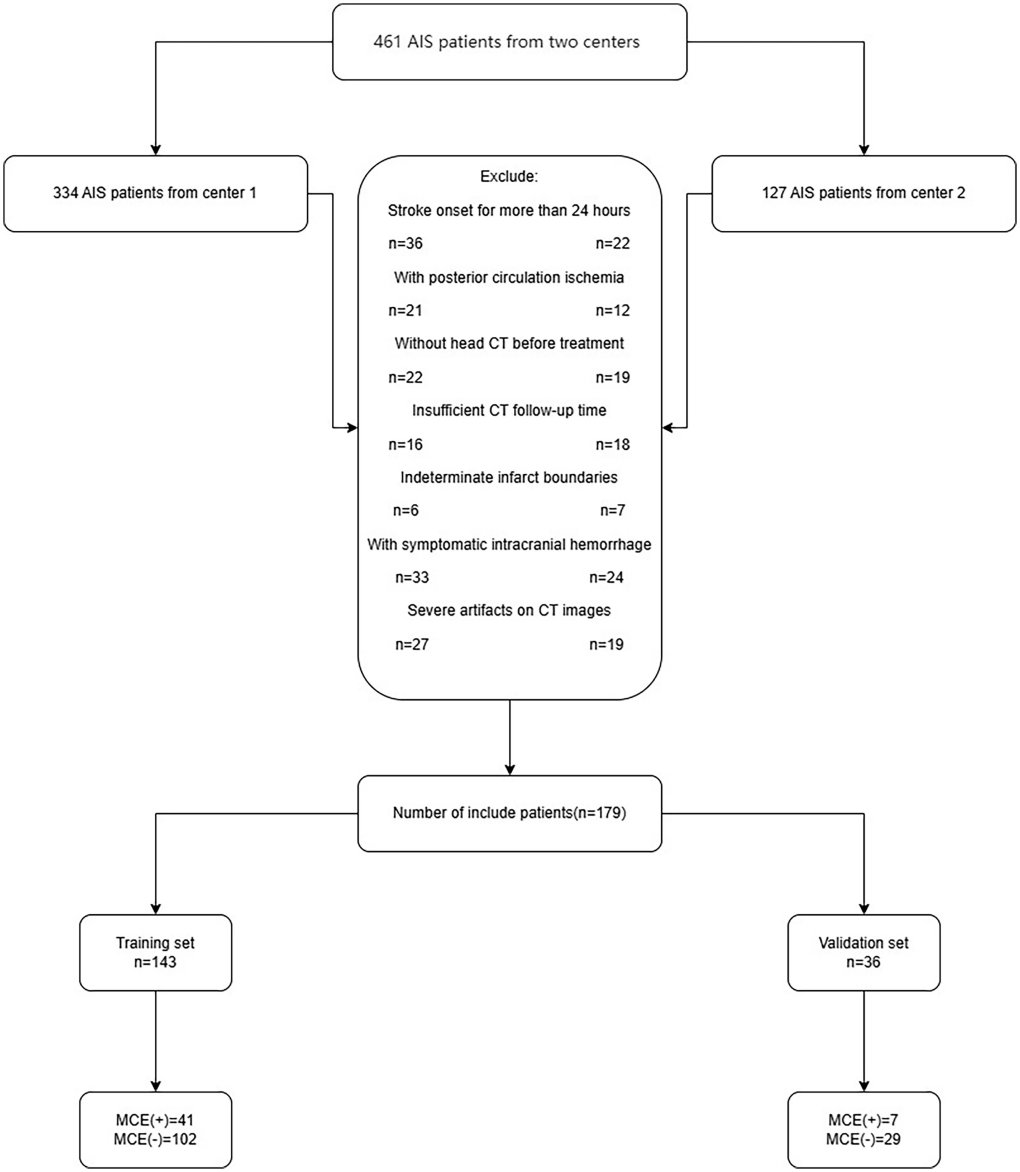
The flow chart of patient inclusion and exclusion criteria. AIS, acute ischemic stroke; MCE, malignant cerebral edema.

Between July 2016 and December 2023, clinical information and NCCT imagery were collected from a pair of hospitals: Affiliated Hospital of North Sichuan Medical College and Chongqing General Hospital. The study collected 461 consecutive AIS patients. The sample included individuals who were diagnosed with AIS and who satisfied the following conditions: (1) anterior circulation affected by AIS as per WHO guidelines (Aho et al., 1980); (2) a head CT scan was conducted within 1 day of symptom emergence before admission for treatment; (3) CT follow-up of at least 3 days unless malignant cerebral edema (MCE) occurred within 3 days; and (4) infarct zones identified by CT. The exclusion criteria were as follows: head trauma, initial brain hemorrhage or tumor, hemorrhagic infarction at admission, post-admission hemorrhagic alteration, insufficient data, and notable irregularities in NCCT imagery. Finally, the study enrolled a total of 179 patients, 131 of whom did not have MCE and 48 of whom did have MCE (Figure 1).

### 2.2 Endpoints

The main endpoint was any midline movement (be it the septum pellucidum or pineal gland) exceeding 5 mm during follow-up examination that required a decompressive hemicraniectomy craniectomy (DHC) (Barber et al., 2003; Tracol et al., 2020).

### 2.3 Data collection

The clinical data in our research included age, sex, stroke onset timing, and treatment, which were collected from the medical records. Additionally, assessments were carried out on patients regarding their NIHSS score; smoking habits; alcohol consumption; hypertension; diabetes; high fat levels; atrial fibrillation; and heart failure. The imaging data in our research included the ASPECTS, severe stroke, and hyperdense middle cerebral artery sign, which was obtained from the Picture Archiving and Communication System (PACS).

### 2.4 Capturing and analyzing images

Our research used the LightSpeed VCT (GE) or IQon Spectral CT (Philips) technique to capture head CT scans without any contrast enhancement. The patients were laid on their backs, with the orbital tract line serving as the baseline for scanning. The scanning zone extended from the apex of the head to the base of the skull. The scanning criteria were as follows: a tube voltage of 120 kV, a current setting of 220 mAs, a pitch ratio of 1.0, and layer thickness of 5 mm and layer spacing of 5 mm.

The patients’ NCCT scans were examined by two seasoned radiologists (F and Y, each with more than 5 years of expertise, independently). (1) The initial CT scan delineated the infarct area boundaries by identifying regions of marked hypointensity following the adjustment of image gray values. (2) The hyperdense middle cerebral artery (MCA) sign exhibited increased densification within the MCA on the affected side of the infarct compared to the contralateral side. (3) Massive cerebral infarction was defined as an infarct area that was more than one-third of the cerebral hemisphere or an infarct volume > 80 mL (Hua et al., 2023). Furthermore, the ASPECTS was determined by subtracting the hypodense area score from the NCCT image’s overall score of 10 (Mokin et al., 2017). Upon admission, the patient’s initial neurological condition was evaluated utilizing the NIHSS. Discrepancies in the assessments made by the two evaluators were settled by mutual agreement via dialog.

### 2.5 Data preprocessing

Missing values in the clinical data were filled using K nearest neighbors (KNN) (Ren et al., 2023). Moreover, normalization of the NCCT images was achieved by adjusting the image voxels to dimensions of 1 mm × 1 mm × 1 mm via linear interpolation, refining the images with a Gaussian filter, and setting the gray bin width of the images at 25.

### 2.6 Infarct lesion segmentation and radiomics feature screening

Infarct lesion segmentation and screening of radiomic feature were performed using 3DSlicer.^1^

**Figure 2:**
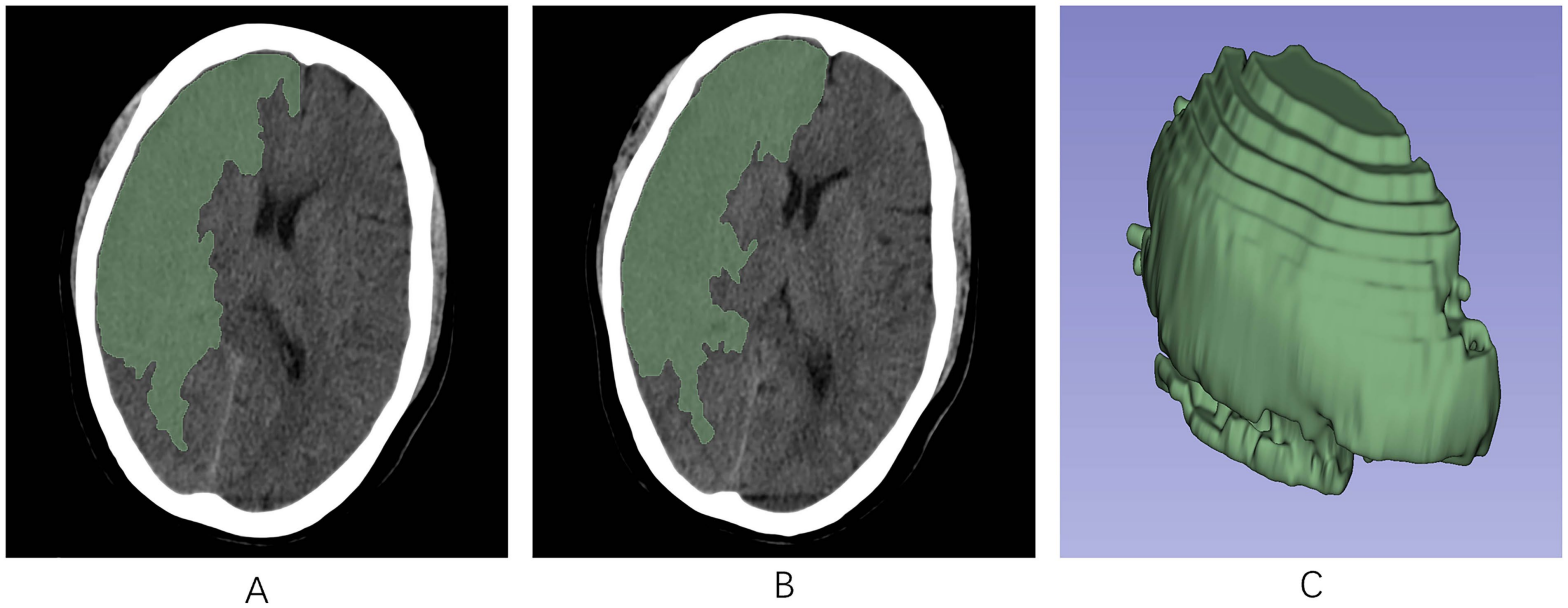
The region of interest (ROI) was manually segmented along the edges of the infarcted region on each slice of the NCCT image using the 3D-Slicer software. **(A,B)** ROIs in the infarct area across different axial planes. **(C)** A 3D-ROI within the infarcted area. ROI, region of interest; 3D, three-dimensional.

This software facilitated the analysis of the initial head CT scans of all AIS patients, which were not enhanced in contrast, in DICOM format. The perimeters of the infarct area were ascertained by modifying the gray hues in the CT scans. Following this, a semiautomatic segmentation technique was employed to acquire a three-dimensional region of interest (ROI) for the infarct area (Figure 2). To maintain consistency, the same radiologist selected 90 AIS patients at random after 1 week for the purpose of resegmentation of the ROIs and extraction of the radiomics features by imaging. The ICC was used to assess the uniformity of characteristics between two occasions. To avoid overfitting, feature selection was conducted as a necessary preprocessing step. Accordingly, only features demonstrating an Intraclass Correlation Coefficient (ICC) value above 0.75 were selected for inclusion in subsequent analyses to ensure their reliability for future studies.

Using the PyRadiomics plug-in of the software, radiomics signature was derived from the ROIs in the images. These included various matrices, such as the gray-level co-occurrence matrix (GLCM), gray-level dependence matrix (GLDM), gray-level run length matrix (GLRLM), gray-level size zone matrix (GLSZM), neighboring gray-tone difference matrix (NGTDM), wavelet-based features, 3D-shaped features, and first-order features.

After analyzing the radiomics features extracted twice using ICC, we subjected the radiomics signature with an ICC > 0.75 to a *t*-test for intergroup comparisons of radiomics scores. Subsequently, LASSO regression and 5-fold cross-validation were employed to identify and ascertain the most suitable radiomics signature linked with the MCE. Lasso regression is a regression analysis technique that has gained considerable recognition for its capacity to identify pivotal features in radiomics, avert overfitting, and yield sparse models that are concise and readily interpretable. By incorporating a regularization term, it reduces model complexity, automatically selects features that contribute the most to the model, and demonstrates robustness against outliers and noise (Huang et al., 2016; Bhatia et al., 2019; Kim et al., 2019). Ultimately, we incorporated the radiomics features associated with MCE obtained after screening and created a radiomics score by a logistic regression algorithm. The Rad-score for every patient on radiomics was calculated by summing the regression coefficients and the product of the equation’s characteristics.

### 2.7 Model building and validation

These patients were assigned to two sets: one for training (*n* = 143) and another for validation (*n* = 36), keeping the ratio at 8:2. In the training cohort, clinical variables were screened using t-test and ANOVA to select variables with *p* < 0.05. According to the one-way analysis, clinical factors with *p* < 0.05, along with Rad-score, were identified as significant risk factors for MCE. Using techniques such as KNN, Support Vector Machine (SVM), Tree, eXtreme Gradient Boosting (XGB), and LR, five distinct machine learning models were developed. The models were then validated in a separate cohort, and their predictive performance was further evaluated. The AUC of the models was determined through the analysis of the subject’s operating characteristic curve. Calibration curves were employed to evaluate the concordance between the model’s predicted probabilities and observed outcomes. Subsequently, clinical decision curve analysis (DCA) was applied to determine the model’s net clinical benefit.

### 2.8 Analytical statistics

Analysis of the statistical data was conducted with R software (version 4.3.1)^2^ and Python 3.9.^3^ Normally distributed data are represented as means ± standard deviation, whereas qualitatively distributed data are represented as numbers and percentages. The evaluation of clinical traits was conducted through the application of t-test and ANOVA. A two-by-two comparative analysis of the AUC across three models was conducted utilizing the DeLong test. In every statistical evaluation, *p* values on both sides below 0.05 were deemed significant.

ICC analyses were conducted to compare radiomics scores between groups. Radiomics features were filtered using a t-test with high correlation and no redundancy. The best feature subset was selected, and the radiomics model was developed through LASSO logistic regression within the ‘Glmnet’ software suite. The ‘class’, ‘kernlab’, ‘rpart’, ‘xgboost’ and ‘glmnet’ packages were utilized to construct KNN, SVM, Tree, XGBoost, and LR machine learning models, respectively. The ‘pROC’, ‘rmda’, and ‘rms’ packages were utilized to construct receiver operating characteristic curve (ROC) curves, DCA curves, and calibration curves, respectively. To evaluate the variance in the AUC among the different models, Delong tests were conducted. Statistical significance was attributed solely to tests where the p value was less than 0.05.

## 3 Results

### 3.1 Clinical demographic characteristics

The study analyzed data from 179 patients (102 males [56.98%] and 77 females [43.02%]) with a median age of 68.89 years from two hospitals. Of these, 48 patients suffered from MCE, while 131 did not. There were no significant differences between the patients’ clinical data (Tables 1, 2). Notable differences were detected in the NIHSS score, infarct volume, and ASPECTS among the MCE (+) and MCE (−) groups within the training dataset after univariate analysis (P<0.001) (Table 3).

**Table 1:** Baseline characteristics in the training and validation cohorts.

Clinical factors	Training cohort (*n* = 143)	Validation cohort (*n* = 36)	*p*-value
Sex			0.102
Female	60 (41.96%)	17 (47.22%)	
Male	83 (58.04%)	19 (52.78%)	
Age Group(years)			0.652
<50	5 (3.5%)	0(0.0%)	
50–60	39 (27.27%)	7 (19.44%)	
60–70	37 (25.87%)	8 (22.22%)	
70–80	34 (23.78%)	14 (38.89%)	
>80	28 (19.58%)	7 (19.44%)	
Smoking			0.752
No	94 (65.73%)	22 (61.11%)	
Yes	49 (34.27%)	14 (38.89%)	
Alcohol			0.491
No	100 (69.93%)	23 (63.89%)	
Yes	43 (30.07%)	13 (36.11%)	
Hypertension			1
No	61 (42.66%)	14 (38.89%)	
Yes	82 (57.34%)	22 (61.11%)	
Diabetes			0.846
No	98 (68.53%)	20(55.56%)	
Yes	45 (31.47%)	16 (44.44%)	
Hyperlipidemia			0.034
No	105 (73.43%)	27 (75.0%)	
Yes	38 (26.57%)	9 (25.0%)	
Atrial fibrillation			0.931
No	84 (58.74%)	23 (63.89%)	
Yes	59 (41.26%)	13 (36.11%)	
Heart failure			1
No	62 (43.36%)	15 (41.67%)	
Yes	81 (56.64%)	21 (58.33%)	
Middle cerebral artery stenosis			1
No	100 (69.93%)	29 (80.56%)	
Yes	43 (30.07%)	7 (19.44%)	
Large area brain infarction			1
No	78 (54.55%)	24 (66.67%)	
Yes	65 (45.45%)	12 (33.33%)	
Treatment			0.497
Non-reperfusion	84 (58.74%)	23 (63.89%)	
IVT	22 (15.38%)	5 (13.89%)	
MT	29 (20.28%)	6 (16.67%)	
IVT with MT	8 (5.59%)	2 (5.56%)	
Age	68.24 (58.5, 78.0)	71.47 (65.75, 78.0)	0.147
Stroke Time	8.6 (2.5, 11.5)	8.72 (4.0, 11.0)	0.935
NIHSS	13.52 (8.0, 18.0)	11.83 (6.75, 16.0)	0.216
Volume	86.83 (28.4, 122.26)	69.63 (16.4, 102.81)	0.235
ASPECTS	6.75 (5.0, 9.0)	7.17 (6.0, 9.0)	0.336

Data are expressed as the mean ± standard deviation, median (interquartile range) or frequency (constituent ratio). IVT, intravenous thrombolysis; MT, mechanical thrombectomy; NIHSS, National Institutes of Health stroke scale score; ASPECTS, Alberta Stroke Program Early CT Score.

**Table 2:** Baseline characteristics of the malignant cerebral edema (MCE) and non-MCE cohorts.

Variable	Total (*n* = 179)	Non-MCE (*n* = 131)	MCE (*n* = 48)	*p*-value
Sex				0.799
Female	77 (43.02%)	54 (41.22%)	23 (47.92%)	
Male	102 (56.98%)	77 (58.78%)	25 (52.08%)	
Age group (years)				0.657
<50	5 (2.79%)	4 (3.05%)	1 (2.08%)	
50–60	46 (25.7%)	31 (23.66%)	15 (31.25%)	
60–70	45 (25.14%)	35 (26.72%)	10 (20.83%)	
70–80	48 (26.82%)	35 (26.72%)	13 (27.08%)	
>80	35 (19.55%)	26 (19.85%)	9 (18.75%)	
Smoking				0.453
No	116 (64.8%)	81 (61.83%)	35 (72.92%)	
Yes	63 (35.2%)	50 (38.17%)	13 (27.08%)	
Alcohol				0.887
No	123 (68.72%)	83 (63.36%)	40 (83.33%)	
Yes	56 (31.28%)	48 (36.64%)	8 (16.67%)	
Hypertension				0.185
No	75 (41.9%)	56 (42.75%)	19 (39.58%)	
Yes	104 (58.1%)	75 (57.25%)	29 (60.42%)	
Diabetes				0.251
No	118 (65.92%)	89 (67.94%)	29 (60.42%)	
Yes	61 (34.08%)	42 (32.06%)	19 (39.58%)	
Hyperlipidemia				1
No	132 (73.74%)	92 (70.23%)	40 (83.33%)	
Yes	47 (26.26%)	39 (29.77%)	8 (16.67%)	
Atrial fibrillation				0.81
No	107 (59.78%)	80 (61.07%)	27 (56.25%)	
Yes	72 (40.22%)	51 (38.93%)	21 (43.75%)	
Heart failure				0.47
No	77 (43.02%)	70 (53.44%)	7 (14.58%)	
Yes	102 (56.98%)	61 (46.56%)	41 (85.42%)	
Middle cerebral artery stenosis				0.743
No	129 (72.07%)	104 (79.39%)	25 (52.08%)	
Yes	50 (27.93%)	27 (20.61%)	23 (47.92%)	
Large area brain infarction				0.529
No	102 (56.98%)	92 (70.23%)	10 (20.83%)	
Yes	77 (43.02%)	39 (29.77%)	38 (79.17%)	
Treatment				0.536
Non-reperfusion	107 (59.78%)	70 (53.44%)	37 (77.08%)	
IVT	27 (15.08%)	24 (18.32%)	3 (6.25%)	
MT	35 (19.55%)	29 (22.14%)	6 (12.5%)	
IVT with MT	10 (5.59%)	8 (6.11%)	2 (4.17%)	
Age	68.89 (59.0, 78.0)	69.45 (60.0, 78.0)	67.35 (57.75, 78.0)	0.3
Stroke time	8.63 (3.0, 11.0)	8.86 (3.0, 12.0)	7.98 (3.0, 10.0)	0.512
NIHSS	13.18 (7.0, 17.5)	11.95 (6.5, 17.0)	16.56 (12.0, 18.25)	0*
Volume	83.37 (24.02, 119.44)	60.56 (12.85, 86.5)	145.62 (92.62, 175.78)	0*
ASPECTS	6.83 (5.0, 9.0)	7.32 (6.0, 9.0)	5.5 (4.0, 7.0)	0*

Data are expressed as the mean ± standard deviation, median (interquartile range) or frequency (constituent ratio). The *P*-value for NIHSS, volume, and aspects is less than 0.001. IVT, intravenous thrombolysis; MT, mechanical thrombectomy; NIHSS, National Institutes of Health stroke scale score; ASPECTS, Alberta Stroke Program Early CT Score.

**Table 3:** Univariate analysis of the risk factors for malignant cerebral edema (MCE) in the training cohort.

	Training cohort (n = 143)		
Clinical factors	MCE (*n* = 41) (28.67%)	Non-MCE (*n* = 102) (71.33%)	*p*-value
Sex			0.465
Female	19 (46.34%)	41(40.2%)	
Male	22 (53.66%)	61(59.8%)	
Age Group(years)			0.376
<50	1 (2.44%)	4 (3.92%)	
50–60	13 (31.71%)	26 (25.49%)	
60–70	8 (19.51%)	29 (28.43%)	
70–80	10 (24.39%)	24 (23.53%)	
>80	9 (21.95%)	19 (18.63%)	
Smoking			0.905
No	31 (75.61%)	63 (61.76%)	
Yes	10 (24.39%)	39 (38.24%)	
Alcohol			1
No	35 (85.37%)	65 (63.73%)	
Yes	6 (14.63%)	37 (36.27%)	
Hypertension			0.345
No	18 (43.9%)	43 (42.16%)	
Yes	23 (56.1%)	59 (57.84%)	
Diabetes			0.515
No	25 (60.98%)	73 (71.57%)	
Yes	16 (39.02%)	29 (28.43%)	
Hyperlipidemia			1
No	34 (82.93%)	71(69.61%)	
Yes	7 (17.07%)	31 (30.39%)	
Atrial fibrillation			1
No	22 (53.66%)	62 (60.78%)	
Yes	19 (46.34%)	40 (39.22%)	
Heart failure			0.702
No	6 (14.63%)	56 (54.9%)	
Yes	35 (85.37%)	46 (45.1%)	
Middle cerebral artery stenosis			1
No	21 (51.22%)	79 (77.45%)	
Yes	20 (48.78%)	23 (22.55%)	
Large area brain infarction			0.818
No	9 (21.95%)	69 (67.65%)	
Yes	32 (78.05%)	33 (32.35%)	
Treatment			0.633
Non-reperfusion	31 (75.61%)	53 (51.96%)	
IVT	3 (7.32%)	19 (18.63%)	
MT	5 (12.2%)	24 (23.53%)	
IVT with MT	2 (4.88%)	6 (5.88%)	
Age, years	67.66 (58.0, 78.0)	68.47 (59.0, 77.75)	0.714
Stroke time	7.85 (3.0, 10.0)	8.9 (2.0, 14.25)	0.491
NIHSS	17.34 (13.0, 19.0)	11.99 (7.0, 17.0)	0*
Volume	146.91 (90.92, 177.49)	62.68 (13.25, 89.09)	0*
ASPECTS	5.51 (4.0, 7.0)	7.25 (6.0, 9.0)	0*

Data are expressed as the mean ± standard deviation, median (interquartile range) or frequency (constituent ratio). The *P*-value for NIHSS, volume, and aspects is less than 0.001. IVT, intravenous thrombolysis; MT, mechanical thrombectomy; NIHSS, National Institutes of Health stroke scale score; ASPECTS, Alberta Stroke Program Early CT Score.

### 3.2 Radiomics score and nomogram development

**Figure 3:**
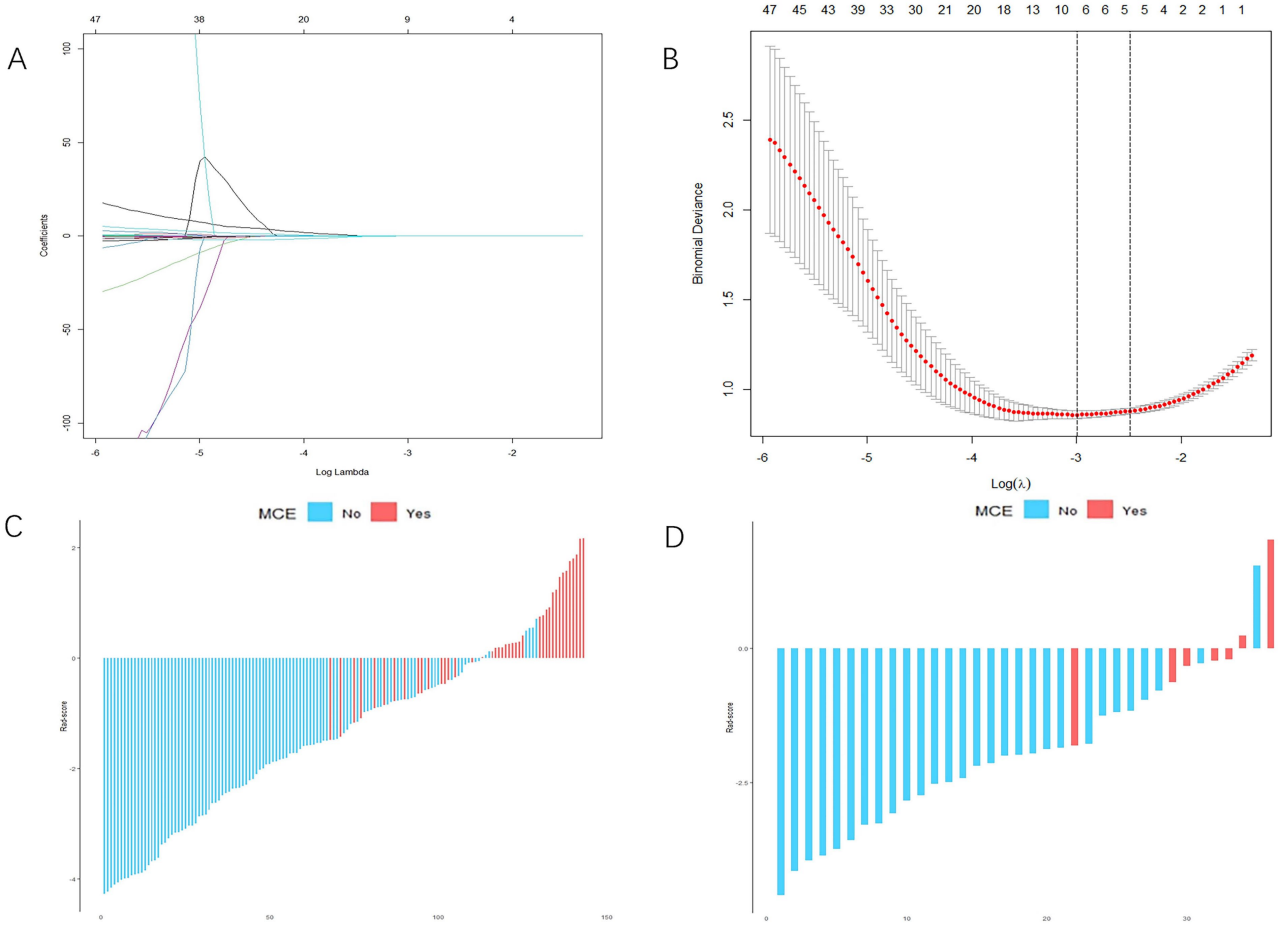
Radiomics score (Rad-score) development. **(A,B)** Radiomics feature selection based on least absolute shrinkage and selection operator (LASSO) and 5-fold cross-validation. When binomial deviance is the smallest, a total of nine optimal features are obtained **(A)** shows the LASSO regression model’s variable reduction and coefficient adjustment. **(B)** Shows the model’s mean squared error (MSE) evolving with the log of the penalty coefficient *λ*, helping to pinpoint optimal features. **(C,D)** The Rad-score of each patient in the training and validation cohorts. Red indicates malignant cerebral edema (MCE), and blue indicates non-MCE.


From each patient, 851 distinct radiomics features were derived, focusing on the specific region of interest (ROI) within the infarct zone, as seen in non-contrast-enhanced CT scans. After conducting an ICC analysis and t-test, we identified 215 stable features with between-group differences. Ultimately, 9 radiomics features showed a strong correlation with the MCE, as determined through LASSO and a 5-fold cross-validation process (Figures 3A,B). The meanings of these 9 radiomics features are listed in Supplementary Table S1. The 9 characteristics were examined using logistic regression to formulate an equation. Subsequently, the Rad-score was determined using the regression coefficients in the formula to demonstrate the predictive accuracy of the MCE.

$$\begin{array}{c} \mathrm{Rad}-\mathrm{Score}=0.15189420\times \mathrm{wavelet}\\ -HLH\_\mathrm{glrlm}.\_\mathrm{LongRunLowGrayLevelEmphasis}\\ +0.11007298\times \mathrm{original}\_\mathrm{glcm}.\_\mathrm{JointAverage}+0.04908581\\ \times \mathrm{original}\_\mathrm{shape}.\_\mathrm{MinorAxisLength}-0.04678184\times \mathrm{wavelet}\\ -HLH\_\mathrm{firstorder}.\_\mathrm{Skewness}+0.01934688\mathrm{wavelet}\\ -HLH\_\mathrm{glszm}.\_\mathrm{SizeZoneNonUniformity}+0.01459792\\ \times \mathrm{wavelet}-LLL\_\mathrm{firstorder}.\_\mathrm{Mean}+0.01241250\\ \times \mathrm{original}\_\mathrm{firstorder}.\_\mathrm{RootMeanSquared}+0.00001356\\ \times \mathrm{original}\_\mathrm{shape}.\_\mathrm{SurfaceArea}+0.00000073\\ \times \mathrm{original}\_\mathrm{gldm}.\_\mathrm{GrayLevelNonUniformity}\text{.} \end{array}$$

**Figure 4:**
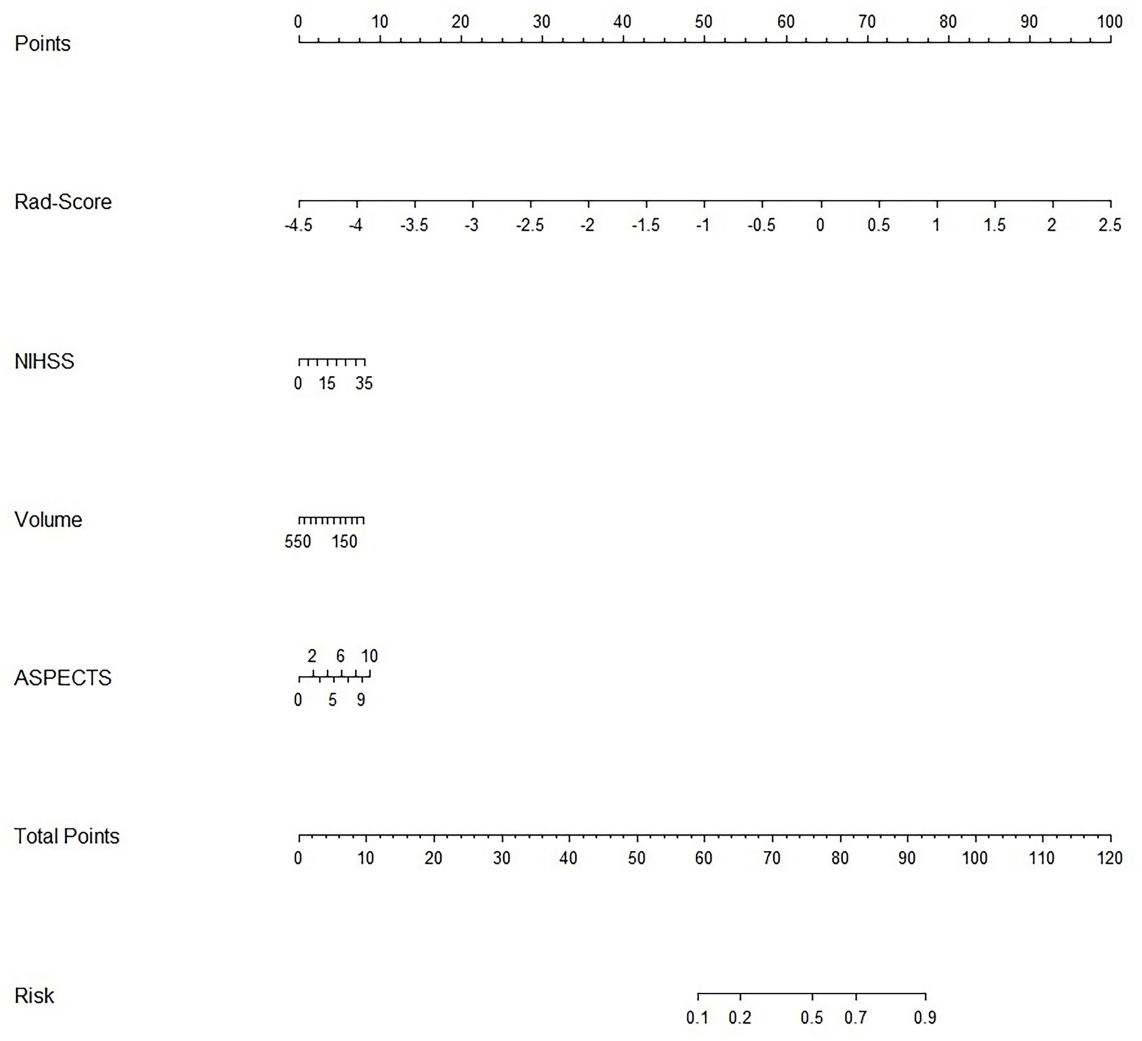
Clinical and radiomics nomogram.


For each patient, waterfall diagrams were generated utilizing the Rad-score (Figures 3C,D). The nomogram was created by combining the clinical risk factors mentioned above, namely, the NIHSS at admission, stroke volume, and ASPECTS, with the radiomics labels. Each best predictor was assigned a separate score, and the total score were greater different between MCE patients and non-MCE patients (mean 0.198 vs. −2.048, *p* < 0.001) (Figure 4). The formula for calculating the risk score is provided.

$$\begin{array}{c} \mathrm{Risk}\_\mathrm{Score}=-0.472296011411395\times \mathrm{Intercept}\\ +1.8696602424284\times RS+0.0304285470958009\\ \times \mathrm{NIHSS}+-0.00188490867690965\\ \times \mathrm{Volume}+0.114446318557212\times \mathrm{ASPECTS} \end{array}$$

### 3.3 Machine learning models development

**Figure 5:**
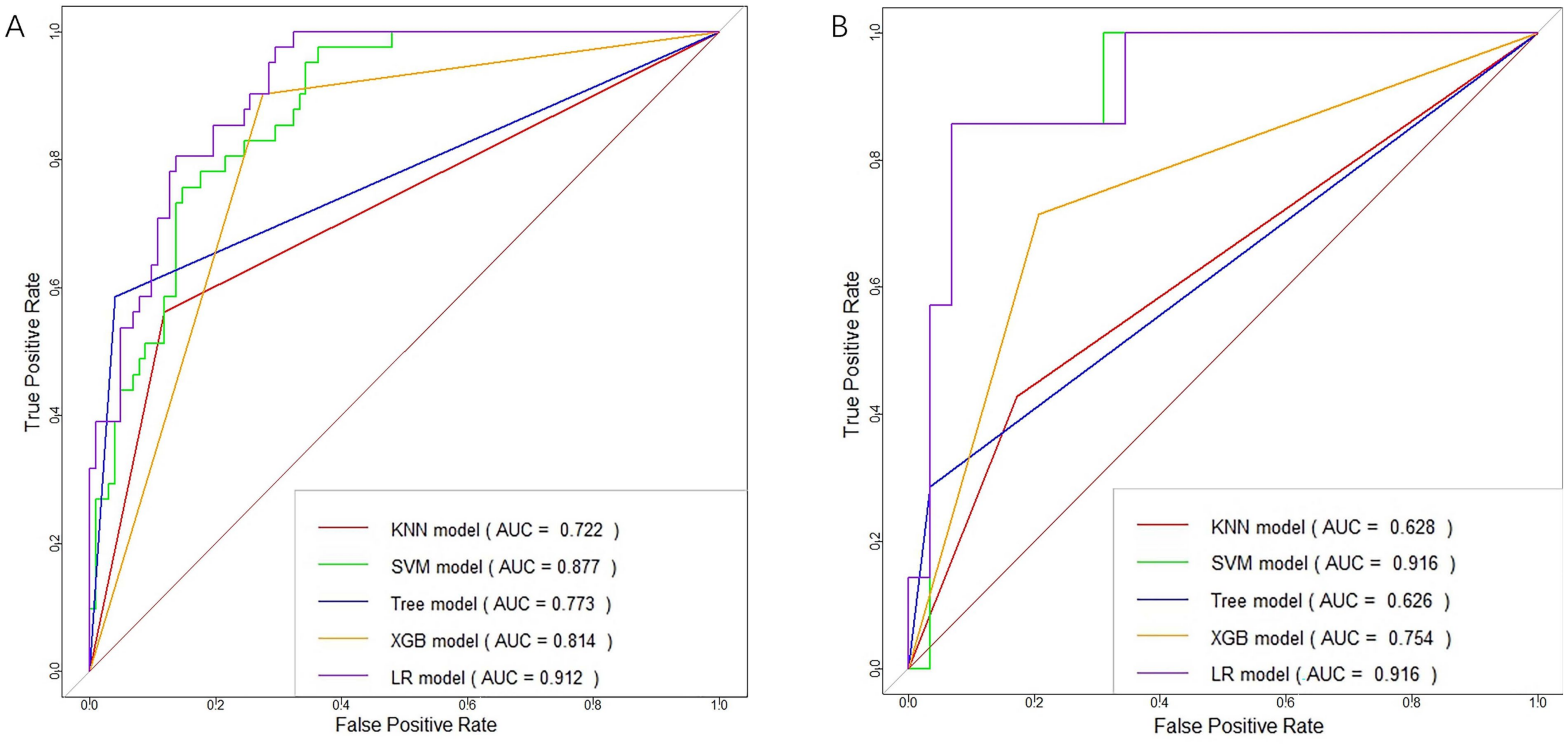
ROC curves of clinical-radiomic features in the training **(A)** and validation **(B)** cohorts for each machine learning model.

The training of the model utilized clinical factors (NIHSS score, infarct volume, ASPECTS), achieving a *p* < 0.05 following univariate analysis and the examination of radiomics features through t-test and logistic regression, utilizing five distinct ML algorithms: KNN, SVM, Tree, XGB, and LR. Model performance was evaluated using the AUCs to identify the optimal machine learning algorithms based on clinical-radiomics features (Table 4, Figures 5A,B). The LR model successfully forecasted the likelihood of MCE, with AUCs of 0.912 (95% CI, 0.868–0.957) and 0.916 (95% CI, 0.808–1.000) in the training and validation cohorts, respectively. Its accuracy, sensitivity, specificity, positive prediction rate and negative prediction rate in the validation set were 0.861, 0.750, 0.875, 0.429, and 0.966, respectively. Despite the low PPV in the validation set, the model can still be considered to have good predictive accuracy when combined with other metrics. Clinical-radiomics machine learning models based on LR were created by incorporating clinical risk factors and key radiomics features from the training cohort, and then validated in the validation cohort. The logistic regression classification algorithm in machine learning was selected for the construction of the clinical model, radiomics model, and joint clinical-radiomics model in the training set and validation set, respectively.

**Table 4:** Diagnostic performance of clinical-radiomic features across machine learning models in training and validation cohorts.

Models	Cohorts	AUC	95% CI	SEN	SPE	ACC
LR model	Training cohort	0.912	(0.868, 0.957)	0.722	0.860	0.825
	Validation cohort	0.916	(0.808, 1.000)	0.750	0.875	0.861
KNN model	Training cohort	0.722	(0.639, 0.805)	0.657	0.833	0.790
	Validation cohort	0.628	(0.418, 0.838)	0.375	0.857	0.750
SVM model	Training cohort	0.877	(0.822, 0.933)	0.833	0.737	0.741
	Validation cohort	0.916	(0.812, 1.000)	0.500	0.824	0.806
Tree model	Training cohort	0.773	(0.694, 0.852)	0.961	0.585	0.853
	Validation cohort	0.626	(0.442, 0.809)	0.966	0.286	0.833
XGB model	Training cohort	0.814	(0.751, 0.877)	1.000	0	0.713
	Validation cohort	0.754	(0.558, 0.949)	1.000	0	0.806

### 3.4 Model evaluation

To evaluate the performance of each model, we created subject operating characteristic curves and calculated the AUC.

#### 3.4.1 Performance of clinical models

**Figure 6:**
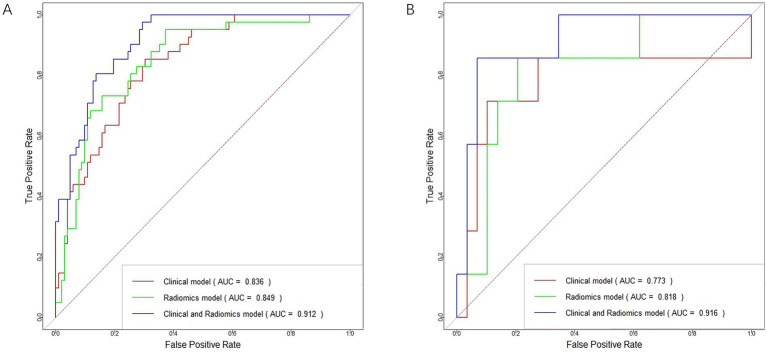
ROC curves for the clinical model, radiomics model, and clinical-radiomics model in the training cohort **(A)** and validation cohort **(B)**.

In distinguishing patients with MCE, the clinical model had an AUC of 0.836 (95% CI, 0.769–0.903) in the training cohort and 0.773 (95% CI, 0.504–1.000) in the validation cohort (Figures 6A,B and Table 5).

**Table 5:** Diagnostic Performance of logistic regression machine learning models based on clinical features, radiomics features, and clinical-radiomics features in training and validation cohorts.

Models	Cohorts	AUC	95% CI	SEN	SPE	ACC
Clinical model	Training cohort	0.836	(0.769, 0.903)	0.667	0.802	0.776
	Validation cohort	0.773	(0.504, 1.000)	0.600	0.871	0.833
Radiomics model	Training cohort	0.849	(0.781, 0.917)	0.667	0.779	0.762
	Validation cohort	0.818	(0.641, 0.994)	0.500	0.824	0.806
Clinical-radiomics model	Training cohort	0.912	(0.868, 0.957)	0.722	0.860	0.825
	Validation cohort	0.916	(0.808, 1.000)	0.750	0.875	0.861

#### 3.4.2 Performance of radiomics models

In terms of discriminating MCE patients, the radiomics model had an AUC of 0.849 (95% CI, 0.781–0.917) in the training cohort and 0.818 (95% CI, 0.641–0.994) in the validation cohort (Figures 6A,B and Table 5).

#### 3.4.3 Performance of combined clinical-radiomics models

**Figure 7:**
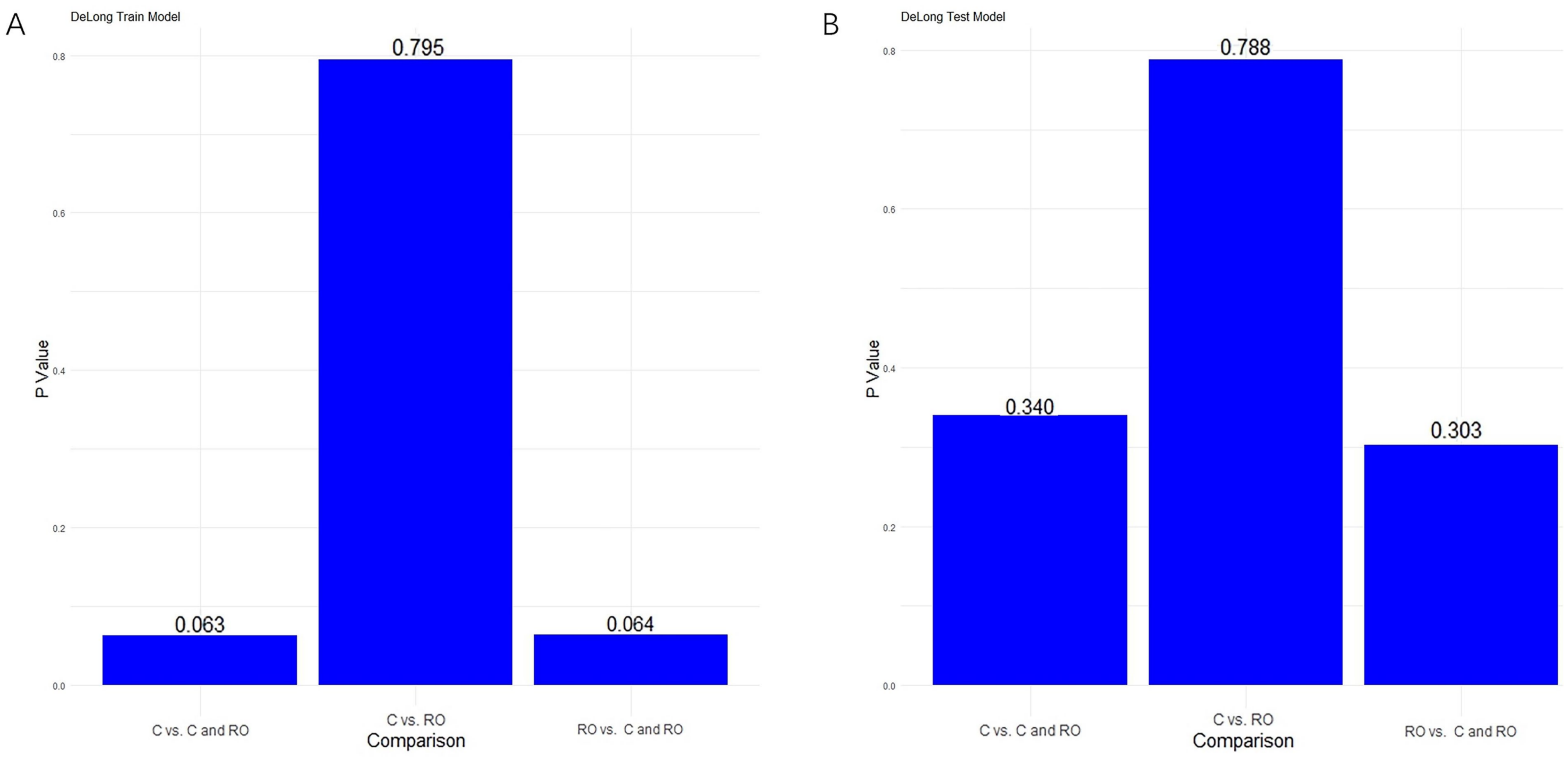
Results of the Delong test in the training cohort **(A)** and validation cohort **(B)**.

For differentiating MCE patients, the combined clinical-radiomics method achieved an AUC of 0.912 (95% CI, 0.868–0.957) in the training cohort and 0.916 (95% CI, 0.808–1.000) in the validation cohort (Figures 6A,B and Table 5). The results from the DeLong test showed no notable statistical variance among three models (*p* > 0.05) in the training and validation cohorts (Figures 7A,B).

**Figure 8:**
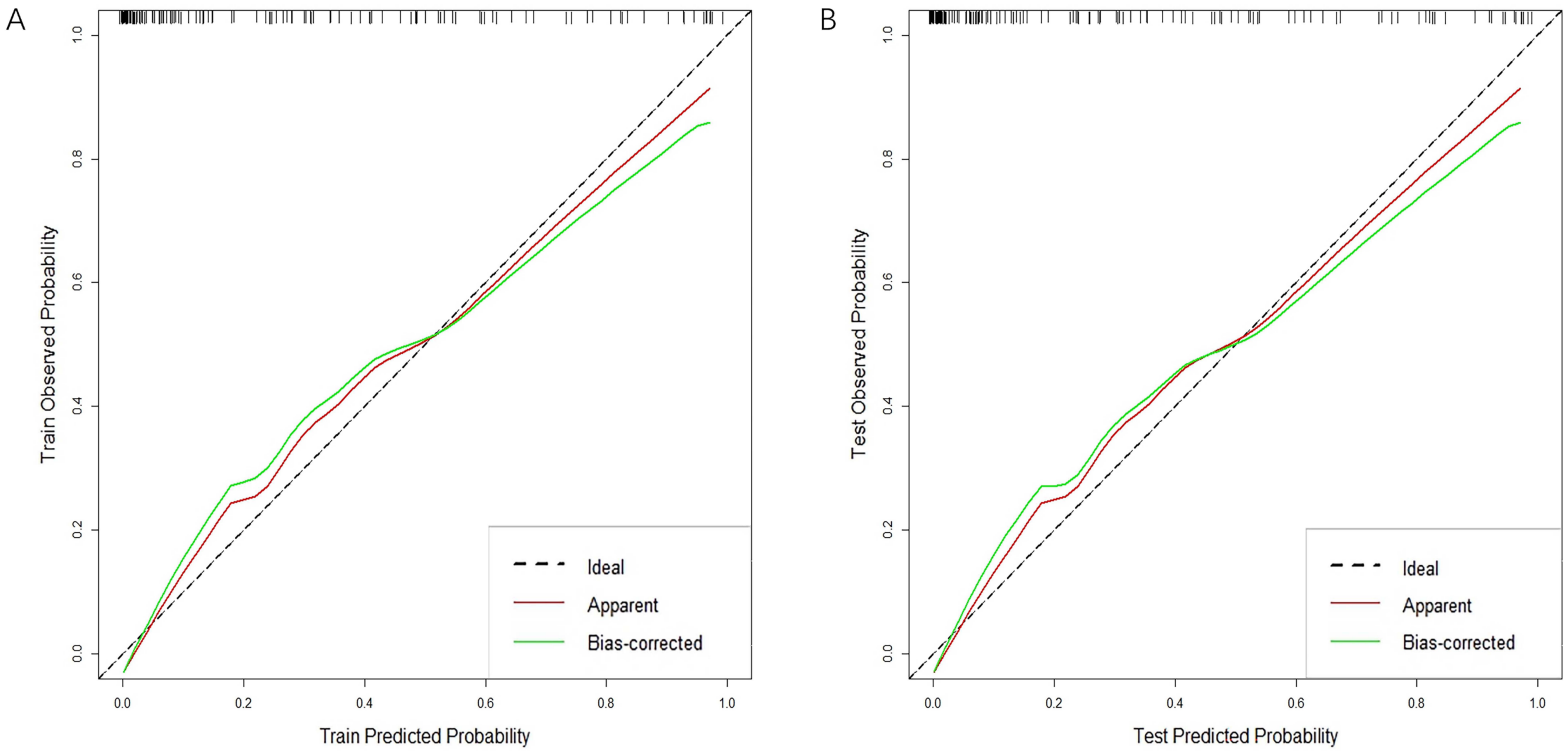
Calibration curves of clinical-radiomics model in the training cohort **(A)** and validation cohort **(B)**.

In both the training and validation sets, there was a strong correlation between the combined clinical-radiomics model and the observed results on the calibration curves for the predictive potential of MCE (Figures 8A,B).

**Figure 9:**
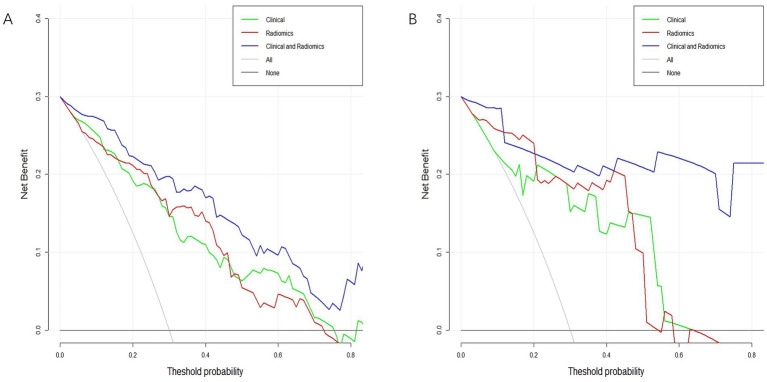
Decision curve analysis curves in the training cohort **(A)** and validation cohort **(B)**.

Analysis of clinical decisions across all three models revealed the clinical-radiomics model’s clinical importance in predicting MCE (Figures 9A,B).

**Figure 10:**
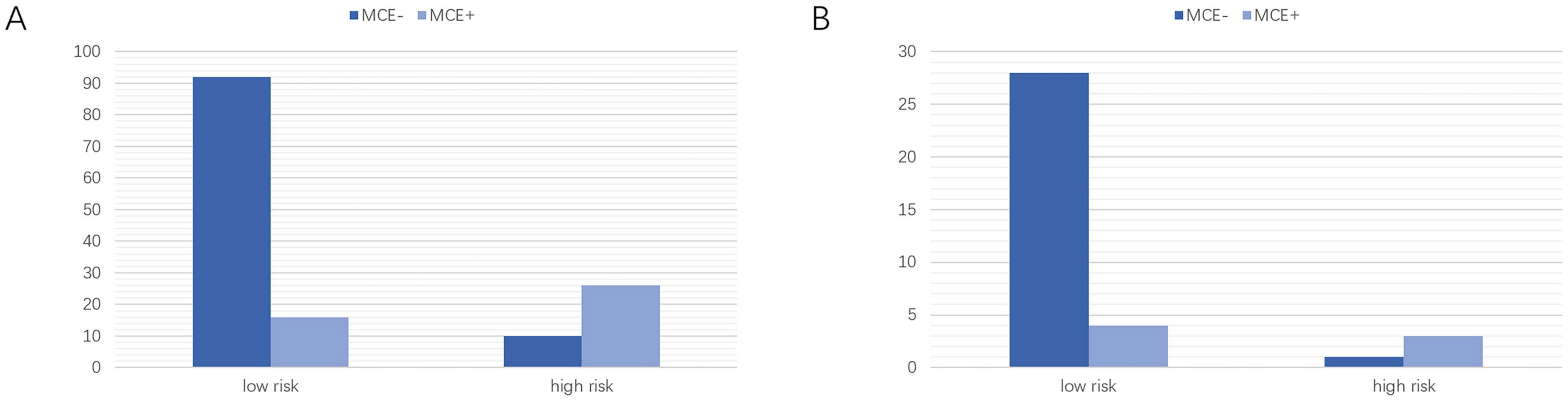
Classification performance of the clinical-radiomics model in the training **(A)** and Validation **(B)** cohorts. MCE, malignant cerebral edema. MCE, malignant cerebral edema.

Based on the optimal critical diagnostic value (0.5) of the clinical-radiomics model, subjects were divided into high-risk and low-risk cohorts in the training and validation sets (Figures 10A,B). The results showed that our model could effectively predict MCE (+).

## 4 Discussion

To our knowledge, the research introduces an innovative machine learning approach to develop a predictive framework for the MCE. This technique combines machine learning techniques with the visualization of radiomics features in infarcted areas using non-contrast-enhanced CT scans and traditional clinical features. This model is capable of evaluating the likelihood of MCE in AIS patients experiencing anterior circulation complications following reperfusion or non-reperfusion treatment. This approach enhances visual recognition in NCCT images for MCE prediction performance and enhances the diagnostic accuracy of primary care doctors. The data were obtained from a multicenter study via routine post admission examinations, allowing for quick and generalizable application of the model. Our model demonstrated AUC values of 0.912 and 0.916 in both training and validation sets, respectively.

Patients suffering from acute ischemic stroke face a critical complication known as MCE, which carries a significant risk of mortality, potentially increasing to 80% (Liebeskind et al., 2019). The usual cause is the sudden blockage of blood vessels in either the proximal middle cerebral artery or the distal internal carotid artery. Cerebral edema is typically a result of the failure of energy-dependent ion transfer from brain cells after AIS and disruption of the blood–brain barrier. These factors combine to allow excess water to leak into brain tissue, impairing normal neuronal function (Hu and Song, 2017; Abdullahi et al., 2018). Prompt identification of MCE and prompt surgical intervention, coupled with early debulking decompression, are key to lowering patient mortality risks (Hofmeijer et al., 2009). Current clinical diagnostic measures for MCE rely on CT-based observations of midline brain deviation or brain herniation formation, which are usually indicators of delayed onset (Pham and Ng, 2024). Therefore, it is crucial to identify early diagnostic markers for MCE.

The correlation between clinical data and MCE was analyzed, revealing that the NCCT-based infarct volume, ASPECTS, and NIHSS score were correlated with MCE. These scores could indicate the extent of ischemic or infarcted lesions. Patients with MCE (+) had lower ASPECTS, greater infarct volume, and higher NIHSS scores than patients with MCE (−). Research indicates that individuals with MCE (+) experienced more intense initial infarct size and higher NIHSS scores than did those with MCE (−), which is consistent with the results of Wu et al. (2018). Furthermore, the research revealed that patients with MCE (+) exhibited a reduced ASPECTS upon admission in comparison to those with MCE (−), which is consistent with the results of Jo et al. (2017). (*p* = 0.007).

Radiomics features indicate the intensity, distribution, and interrelationships between pixels that cannot be observed by the naked eye. Radiomics feature analysis is widely used in radiomics to convert high-throughput data extracted from medical images into quantitative metrics. These metrics provide a better and more intuitive understanding of disease heterogeneity (Castellano et al., 2004; Miles et al., 2013; Ren et al., 2018). Currently, there are relatively few applications of imaging in AIS. CT-based radiomics has proven effective in predicting hemorrhagic transformation risks in AIS patients and in evaluating the prognosis of those undergoing standard treatment (Zhang et al., 2023; Heo et al., 2024). Furthermore, radiomics analysis using MRI successfully pinpointed the time of stroke onset in AIS patients (Lu et al., 2024). Additionally, the radiomics features of brain MRI images could serve as initial indicators for the emergence of cognitive deficits and pneumonia poststroke (Luo et al., 2024; Wang et al., 2024). This study presents a radiomics model that uses signature extracted from NCCT images to predict serious complications-MCE after AIS. The model performed well, and the 9 best quantitative radiomics signature were identified. Among them, the GLRLM feature reflects the distribution of consecutive low gray pixels in the image, one GLCM feature reflects the homogeneity of the image texture, two shape features reflect the shape properties of the image region, three first-order features, one GLSZM feature reflects the roughness of the image texture, and one GLDM feature reflects the similarity of the image dependencies.

Among them, the wavelet transform-based grayscale tour length matrix (GLRLM) feature (wavelet-HLH_glrlm._LongRunLowGrayLevelEmphasis) and the grayscale covariance matrix (GLCM) (original_glcm._JointAverage) were identified as the most important features. Ischemic stroke is an injury to brain tissue due to cerebrovascular obstruction in which dysfunction of certain ion channels and transporters can cause disruption of the blood–brain barrier. This disruption can lead to edema and structural changes in the brain tissue of the affected region. Conventional imaging techniques, such as computed tomography (CT) and magnetic resonance imaging (MRI), are primarily used to identify areas of cerebral infarction, but these techniques typically provide image information only about macroscopic structural changes in the brain, and it is difficult to detect microtextural changes. Radiomics methods are able to reflect information about the texture, density, and shape of brain tissue by extracting high-dimensional features from CT or MRI images, thus providing a finer representation of the microscopic changes in brain tissue after stroke. In imaging, infarcted areas tend to have low gray levels, while surrounding healthy brain tissue has higher gray levels. As a result, there is usually a significant textural difference between the infarcted area and the healthy brain tissue. In this study, the wavelet-HLH_glrlm._LongRunLowGrayLevelEmphasis feature was extracted by wavelet transform (HLH direction) and gray-level run-length matrix (GLRLM) analysis and used to characterize the texture of dark regions in the image. This radiomics feature helps to quantify the heterogeneity between infarcted and normal tissue and thus assess the complexity of the lesion area. For example, high values of this feature indicate a continuous distribution of low gray values over a large area, which may be associated with extensive ischemic necrosis, indicating the severity of brain injury. Another feature, original_glcm_JointAverage, is a texture feature based on a gray value covariance matrix that quantifies the gray value variability of a stroke region by capturing the average relationship of gray values between adjacent pixels. This variability may be related to the severity of the lesion and the homogeneity of the tissue damage. In the present study, the above two types of radiomics features were most significantly correlated with MCE. The predictive performance of the developed radiomics model was better than that of the conventional clinical model (AUC: 0.849 vs. 0.836), suggesting that the model can be used to assess the severity of the lesion and the homogeneity of the tissue damage, to analyze the risk of occurrence of MCE after AIS, and may be helpful in optimizing the therapeutic regimen. These findings indicate that a heightened risk of MCE is correlated with diversity in the stroke area. However, additional studies are required to clarify the connection between MCE and pathological changes in the radiomics features of NCCT-based images. In this research, predictive models developed with the 9 radiomics features closely linked to MCE exhibited notably greater AUCs than those utilizing clinical data in both the training and validation sets. This indicates that radiomics signature surpass clinical records in predicting MCE efficacy. Thus, while infarct volume, the ASPECTS, and the NIHSS score can predict the risk of MCE, models based on NCCT radiomics can further improve MCE prediction performance. This allows clinicians to identify AIS patients requiring debulking decompression at an early stage, thereby improving patient benefit rates.

In this research, the DeLong test did not show statistical significance for the three models. Notably, the clinical-radiomics model demonstrated superior AUC and precision compared to the clinical model across both the training and validation cohorts. This suggests that radiomics has the potential to improve MCE prediction in the future. Additionally, the DCA results demonstrated that this clinical-radiomics model provides a significant net benefit in predicting MCE. Therefore, the clinical-radiomics model can be considered a reliable and reproducible tool to aid treatment decisions. It may be implemented in clinical practice following validation in a larger cohort.

It is crucial to acknowledge the constraints inherent in this research. Initially, the backward-looking aspect of this subject could lead to biases in information and selection. Although the five machine learning algorithms used in this study, namely KNN, Tree, XGB, SVM, and LR, all include strategies for dealing with data imbalance, the applicability of the models still needs to be further confirmed through prospective and multi-center validation with a broader range of samples due to the data set imbalance caused by sample size limitations and the lack of external validation for the constructed models. Additionally, additional studies using CT or Magnetic Resonance Imaging (MRI) brain perfusion imaging might be required for more significant outcomes, given the model’s potential inapplicability to hyperacute AIS patients whose infarct limits are indeterminable by modifying image gray values. Despite its limitations, this study developed a prediction model for MCE by combining radiomics features with clinical features. The proposed model could assist in the prompt and precise prediction of MCE in individuals suffering from acute anterior circulation infarction.

## 5 Conclusion

In summary, this research offers fresh perspectives on forecasting MCE in cases of ischemic stroke. The findings indicate that the integration of clinical and radiomics signature in machine learning models can precisely predict MCE, aiding in clinical decision-making processes.

## Data Availability

The original contributions presented in the study are included in the article/Supplementary material, further inquiries can be directed to the corresponding author.

## Footnotes

^1^https://www.slicer.org/

^2^https://www.r-project.org/

^3^https://www.python.org
